# Dapagliflozin, metformin, monotherapy or both in patients with metabolic syndrome

**DOI:** 10.1038/s41598-021-03773-z

**Published:** 2021-12-20

**Authors:** Lan Cheng, Qianyu Fu, Longhua Zhou, Yuqin Fan, Fenfen Liu, Yuanyuan Fan, Xin Zhang, Weiqing Lin, Xiaohe Wu

**Affiliations:** Jiangxi Provincial People’s Hospital Affiliated to Nanchang University, Nanchang, 330006 Jiangxi China

**Keywords:** Endocrine system and metabolic diseases, Metabolic disorders

## Abstract

The present study evaluated the effects of dapagliflozin, a SGLT2 inhibitor, or dapagliflozin plus metformin versus metformin monotherapy in patients with metabolic syndrome. This study included patients who admitted in Jiangxi Provincial People’s Hospital from January 1, 2017 to December 31, 2019 and were diagnosed with metabolic syndrome. A total of 248 participants were randomly assigned to divide into three groups: dapagliflozin group; metformin group; dapagliflozin in combined with metformin group. Dapagliflozin group and metformin group were associated with similar improvements in components of metabolic syndrome. Relative to dapagliflozin or metformin monotherapy, dapagliflozin combined with metformin provided greater improvements in components of metabolic syndrome. So did HOMA-IR scores, fasting plasma insulin and inflammatory indicators (hsCRP, PMN/HDL-C and Monocytes/HDL-C). Dapagliflozin improved all components of metabolic syndrome in patients with metabolic syndrome. Furthermore, dapagliflozin combined with metformin showed more meaningful improvements in any of components of metabolic syndrome than dapagliflozin or metformin monotherapy.

## Introduction

Metabolic syndrome is a gathering of various metabolic disorders which includes hyperglycemia, central obesity, dislipidaemia and hypertension^1^. In China, the age-standardised incidence rate of metabolic syndrome was 9.8% in males and 17.8% in females^2^. The patients with metabolic syndrome have 2–6 times higher prevalence of developing cardiovascular disease and type 2 diabetes mellitus than those without metabolic syndrome^3^. Insulin resistance plays an important role in the pathogenesis of metabolic syndrome^4^.

Sodium–glucose co-transporter-2 (SGLT-2) inhibitors are novel therapeutic agents for type 2 diabetes mellitus by inhibiting the glucose reabsorption in renal proximal tubules. They differ from other hypoglucemic agents in removing glucose from body, thus reducing system and cellular glucose toxicity^5^. A series of clinical trials^6–8^ have confirmed that SGLT2 inhibitors could improve glycemic control, reduce body weight and attenuate blood pressure which suggested that they may be effective to ameliorate metabolic syndrome.

The present study investigated the effects of dapagliflozin, a SGLT2 inhibitor, or dapagliflozin plus metformin versus metformin monotherapy in patients with metabolic syndrome.

## Methods

### Study design and patient populations

This study included patients who admitted in Jiangxi Provincial People's Hospital from January 1, 2017 to December 31, 2019. There are several definitions of metabolic syndrome described by various professional medical organizations. In this paper, we adopted the definition of the International Diabetes Federation (IDF) as shown below: central obesity (waist circumference > 90 cm in men, or > 80 cm in women) plus met ≥ 2 of the following criteria: triglycerides ≥ 1.7 mmol/L; HDL-C < 1.0 mmol/L in men or < 1.3 mmol/L in women; diagnosis of hypertension or meeting BP-related criteria (systolic BP ≥ 130 mmHg or diastolic BP ≥ 85 mmHg); Impaired fasting glucose (fasting glucose ≥ 5.6 mmol/L). A total of 248 participants were randomly assigned to receive one of three interventions for 1 year: (1) dapagliflozin 10 mg once daily; (2) metformin 850 mg twice daily; (3) dapagliflozin 10 mg once daily in combined with metformin 850 mg twice daily.

The protocol was approved by the ethics committee of Jiangxi Provincial People’s Hospital. The study were conducted in accordance with the ethical principles outlined in the Declaration of Helsinki. Participants provided informed written consent prior to enrollment in this study.

### Endpoints

We have taken similar clinical endpoints in the research conducted by Davies et al.^9^. The details as below: Changes from baseline in glycemic parameters (HbA1c, fasting plasma glucose [FPG] and fasting plasma insulin, HOMA-IR score), anthropometric parameters (body weight, body mass index [BMI], and waist circumference), BP (systolic and diastolic), and lipids (low-density lipoprotein cholesterol [LDL-C], HDL-C, and triglycerides) were assessed at 1 year. HOMA-IR score was calculated using the HOMA-IR formula (HOMA-IR = fasting insulin (mIU/L) × fasting glucose (mmol/L)/22.5. hsCRP and blood cell count were measured in the clinical laboratory of the hospital.

### Statistical analyses

Baseline characteristics are expressed as the number of observations for categorical variables or mean ± standard deviation for continuous variables. Differences among groups were accessed by using one-way ANOVA for continuous variables and the χ^2^ test for categorical variables. For the clinical endpoints (change in various indices from baseline to one year), the analysis of covariance was used. The results were expressed as adjusted mean (standard error). Analysis of covariance included the baseline value of each analyzed variable as covariates. SPSS17.0 statistical software was used. *P* < 0.05 was statistically significant.

## Results

In this study, two hundred and forty-eight patients completed the study and 89, 82, 77 patients in the dapaglifozin group, metformin group, and dapaglifozin plus metformin group, respectively. The baseline clinical characteristics are summarized in Table 1. Most patients were middle aged (average age, approximately 52 years) and overweight (average BMI, approximately 26 kg/m^2^) with moderate hyperglycemia (average HbA1c level, approximately 6.0%). Three groups did not differ regarding any baseline clinical characteristic and there was no difference among the medications in groups.

**Table 1:** Baseline characteristics.

Characteristic	Dapagliflozin group (n = 89)	Metformin group (n = 82)	Dapagliflozin plus metformin group (n = 77)	*P* value
Sex (male)	47 (52.8%)	42 (51.2%)	46 (59.7%)	0.519
Age (years)	50.3 ± 8.2	52.7 ± 9.6	54.1 ± 10.5	0.374
Body weight (kg)	72.6 ± 10.4	75.3 ± 13.2	73.5 ± 12.8	0.669
BMI (kg/m^2^)	26.7 ± 4.1	26.3 ± 4.7	26.5 ± 5.9	0.535
Waist circumference (cm)	99 ± 9.2	101 ± 9.8	102 ± 10.7	0.362
Systolic blood pressure (mmHg)	142 ± 11.4	147 ± 10.9	145 ± 14.8	0.647
Diastolic blood pressure (mmHg)	80 ± 12.7	82 ± 14.6	81 ± 12.8	0.669
Fasting plasma glucose (mmol/L)	6.2 ± 1.5	6.4 ± 1.1	6.3 ± 0.9	0.732
Fasting plasma insulin (mIU/L)	21.4 ± 3.7	23.6 ± 3.6	20.4 ± 4.3	0.571
HOMA-IR	5.9 ± 0.4	6.1 ± 0.5	6.3 ± 0.6	0.315
HbA1c (%)	6.0 ± 0.6	5.9 ± 0.5	6.1 ± 0.7	0.447
HDL cholesterol (mmol/L)	0.9 ± 0.4	0.8 ± 0.3	0.9 ± 0.3	0.642
LDL cholesterol (mmol/L)	3.7 ± 0.9	3.2 ± 0.6	3.4 ± 0.8	0.583
Triglycerides (mmol/L)	2.1 ± 1.0	2.2 ± 1.3	2.0 ± 0.9	0.792
hsCRP (mg/L)	3.2 ± 1.1	3.4 ± 1.6	3.4 ± 1.5	0.365
PMN/HDL-C	0.12 ± 0.06	0.13 ± 0.04	0.12 ± 0.04	0.416
Monocytes/HDL-C	0.11 ± 0.07	0.13 ± 0.07	0.13 ± 0.06	0.582
Current smoking	25 (28.1%)	23 (28.0%)	16 (20.8%)	0.479
**Antihypertensive drugs**				
Diuretic drugs	7 (7.9%)	10 (12.2%)	9 (11.7%)	0.599
Calcium channel blockers	21 (23.6%)	22 (26.8%)	15 (19.5%)	0.549
ACEI/ARB	20 (22.5%)	21 (25.6%)	13 (16.9%)	0.404
β-blockers	4 (4.5%)	6 (7.3%)	4 (5.2%)	0.711
**Lipid-lowering agents**				
Statins	8 (9.0%)	10 (12.2%)	9 (11.7%)	0.769
Fibrates	3 (3.4%)	2 (2.4%)	5 (6.5%)	0.398

Dapagliflozin group and metformin group were associated with similar reductions in components of metabolic syndrome (body weight, BMI, waist circumference, fasting plasma glucose, triglycerides). Relative to dapagliflozin or metformin monotherapy, dapagliflozin combined with metformin provided greater reductions in components of metabolic syndrome (body weight, BMI, waist circumference, fasting plasma glucose, triglycerides). So did HOMA-IR scores, fasting plasma insulin and inflammatory indicators (hsCRP, PMN/HDL-C and Monocytes/HDL-C). Dapagliflozin and metformin monotherapies were associated with similar increasements in HDL-cholesterol and LDL-cholesterol, respectively, and dapagliflozin combined with metformin therapy provided greater increasement in HDL-cholesterol. The reductions in systolic blood pressure and diastolic blood pressure were significantly more pronounced in the dapaglifozin group and dapagliflozin plus metformin group than that in the metformin group. Data shown in Table 2. After 1 year intervention, metabolic syndrome remitted in 41/89 (46.1%) patients, 47/82 (57.3%) patients, and 59/77 (76.6%) patients in the dapagliflozin group, metformin group, and combination treatment group, respectively.

**Table 2:** Summary of clinical endpoints.

Characteristic	Dapagliflozin group (n = 89)	Metformin group (n = 82)	Dapagliflozin plus metformin group (n = 77)
∆Body weight (kg)	− 3.7 (0.8)	− 3.6 (0.7)	− 6.9 (0.9)*^#^
∆BMI (kg/m^2^)	− 1.0 (0.1)	− 1.0 (0.1)	− 1.7 (0.1)*^#^
∆Waist circumference (cm)	− 3.1 (1.0)	− 3.0 (0.8)	− 4.7 (0.9)*^#^
∆Systolic blood pressure (mmHg)	− 3.4 (1.0)^∮^	− 1.1 (0.8)	− 4.2 (0.9)^#^
∆Diastolic blood pressure (mmHg)	− 2.0 (1.0)^∮^	− 0.6 (0.1)	− 2.9 (0.9)^#^
∆Fasting plasma glucose (mmol/L)	− 1.2 (0.1)	− 1.0 (0.1)	− 2.3 (0.2)*^#^
∆Fasting plasma insulin (mIU/L)	− 1.0 (0.8)	− 1.2 (0.8)	− 2.5 (1.1)*^#^
∆HOMA-IR	− 1.5 (1.3)	− 1.4 (1.1)	− 3.1 (1.2)*^#^
∆HbA1c (%)	− 0.8 (0.1)	− 0.8 (0.2)	− 1.3 (0.2)*^#^
∆HDL cholesterol (mmol/L)	0.05 (0.01)	0.04 (0.01)	0.10 (0.02)*^#^
∆LDL cholesterol (mmol/L)	0.07 (0.01)	0.06 (0.01)	0.08 (0.01)
∆Triglycerides (mmol/L)	− 0.69 (0.03)	− 0.86 (0.02)	− 1.9 (0.04)*^#^
∆hsCRP (mg/L)	− 1.0 (0.6)	− 1.2 (0.8)	− 2.5 (1.0)*^#^
∆PMN/HDL-C	− 0.02 (0.02)	− 0.01 (0.02)	− 0.06 (0.02)*^#^
∆Monocytes/HDL-C	− 0.03 (0.04)	− 0.04 (0.04)	− 0.09 (0.03)*^#^
Patients with remitting metabolic syndrome, n (%)	41 (46.1%)	47 (57.3%)	59 (76.6%)*^#^

*Dapagliflozin plus metformin group versus dapagliflozin group, P < 0.05.

^#^Dapagliflozin plus metformin group versus metformin group, P < 0.05.

^∮^Dapagliflozin group versus metformin group, P < 0.05.

## Discussion

Dapagliflozin improved metabolic indicators in patients with metabolic syndrome. Furthermore, dapagliflozin combined with metformin showed more significant improvements in any of indicators of metabolic syndrome than dapagliflozin or metformin monotherapy. Protective effects of dapagliflozin against metabolic syndrome are mostly attributable to the mechanism of action for dapagliflozin. Inhibition of SGLT-2 increases the excretion of glucose in urinary and decreases the level of glucose in plasma, resulting in a osmotic diuresis and caloric loss. Therefore, dapagliflozin confers glycemic improvement, weight loss, and BP reduction^10^.

Davies et al.^9^ and Fuchigami et al.^11^ addressed that canagliflozin or dapaglliflozin improved all components of metabolic syndrome versus glimepiride or sitagliptin. Given that glimepiride and sitagliptin have the neutral effects on metabolic syndrome other than glycemic control, it is notable that we have taken metformin as control in our research which lowered metabolic syndrome prevalence by 17% compared with placebo^12^. Our results further confirmed that the combination of dapagliflozin with metformin has a meaningful impact on metabolic syndrome.

The research conducted by González-Ortiz et al.^10^ have shown that there were more patients (58.3%) with metabolic syndrome in remission in dapagliflozin group than those (46.1%) in our research. The reason may be the difference of the mean age of patients between two studies because an older age in patients is often companied by a more severe disease condition. Another reason may be the difference of the number of patients between two studies because we had 89 patients in dapagliflozin group, while they had only 12 patients. Obviously, there was a more sufficient evidence grade in our research.

The research conducted by Henry et al.^13^ has compared dapagliflozin plus metformin, dapagliflozin monotherapy, and metformin monotherapy in patients with type 2 diabetes. The results consistent with our study were combination treatment had a meaningfully greater reductions in HbA1C, fasting plasma glucose and weight compared with either monotherapy. However, the results inconsistent with our study were they have found that dapagliflozin alone was more effective in reducing fasting plasma glucose and weight than metformin alone, while our study found that dapagliflozin alone was not predominant over metformin alone in reductions of fasting plasma glucose and weight. The possible reason between the difference of two studies may be they enrolled the patients diagnosed with type 2 diabetes, and we enrolled the patients diagnosed with metabolic syndrome.

Because metabolic syndrome is highly associated with insulin resistance, many investigators believe that insulin resistance mediates the metabolic syndrome risk factors^14^. Insulin resistance as reflected by increased fasting plasma insulin and HOMA-IR were showed to be improved in each of three groups and we found that dapagloflizin in combination with metformin had more improvements in fasting plasma insulin and HOMA-IR than monotherapy, suggesting that dapagliflozin combined with metformin had been more effective in insulin resistance status. Inflammation is an another pathogenesis of the metabolic syndrome^15^. hsCRP is the best biomaker in patients with metabolic syndrome^16^. Its plasma level is correlated with the number of metabolic disorders^17,18^ and is correlated with the severity of metabolic syndrome^19^. The opinion that hsCRP is a predictor of cardiovascular events in the future in patients with metabolic syndrome is well-accepted^15^. More recently, Jialal et al. have addressed that neutrophil and monocyte ratios to high-density lipoprotein-cholesterol are better predictors of metabolic syndrome than hsCRP alone^19^. Therefore, we accessed the different effects of three interventions on these inflammatory indictors and had similar results that the three inflammatory indictors were showed to be improved in each group and dapagloflizin in combination with metformin had more improvements than monotherapy, suggesting that dapagliflozin combined with metformin had been more effective in anti-inflammation effects.

Several studies^10,13,20^ evaluated the beneficial effects of SGLT-2 inhibitors in patients with metabolic syndrome or type 2 diabetes. However, most of these studies were performed in a small number of cases or a short follow-up period. Since our study was one of the longer follow-up studies and had the higher number of cases in similar studies so far, the present study evaluated the effects of long-term administrations of dapagliflozin, metformin, or both on metabolic syndrome.

The rational for combining dapagliflozin with metformin as initial treatment for diabetic therapy has been addressed in Guidelines of the American Association of Clinical Endocrinologists^21^ and the Canadian Diabetes Association^22,23^. However, the current study was one of few studies which evaluated the effects of dapagliflozin plus metformin on anthropometric parameters or metabolic parameters in patients with metabolic syndrome. Because the combination therapy showed the beneficial effects and advantages over either monotherapy in patients with metabolic syndrome, our study supported the opinion that a combination therapy of dapagliflozin with metformin should be an initial therapy of metabolic syndrome.

The treatment of metabolic syndrome is a challenging condition. Because dapagliflozin has positive effects on multiple parameters, it may be special effective in patients with metabolic syndrome and ultimately reduces cardiovascular events and mortality in those. Combination dapagliflozin with metformin therapy is more beneficial. Further investigations of the safety with the combination treatment are still required.
